# Nomogram for the cumulative live birth in women undergoing the first IVF cycle: Base on 26, 689 patients in China

**DOI:** 10.3389/fendo.2022.900829

**Published:** 2022-08-25

**Authors:** Pengfei Qu, Lijuan Chen, Doudou Zhao, Wenhao Shi, Juanzi Shi

**Affiliations:** ^1^ Translational Medicine Center, Northwest Women’s and Children’s Hospital, Xi’an, China; ^2^ The NCH Key Laboratory of Neonatal Diseases, National Children’s Medical Center, Children’s Hospital of Fudan University, Shanghai, China; ^3^ Assisted Reproduction Center, Northwest Women’s and Children’s Hospital, Xi’an, China

**Keywords:** cumulative live birth, prediction model, nomogram, IVF, Chinese population

## Abstract

**Objective:**

Predictive models of the cumulative live birth (CLB) in women undergoing *in vitro* fertilization (IVF) treatment are limited. The aim of this study was to develop and validate a nomogram for the CLB in women undergoing the first IVF cycle.

**Methods:**

Based on a cross-sectional study in assisted reproduction center of Northwest Women’s and Children’s Hospital, 26,689 Chinese patients who underwent IVF treatment was used to develop and validate a prediction model for the CLB. Among those participants, 70% were randomly assigned to the training set (18,601 patients), while the remaining 30% were assigned to the validation set (8,088 patients). A nomogram was constructed based on the results of the multivariate logistic regression analysis. The model performance was evaluated using the C statistic and the calibration performance was assessed by Hosmer-Lemeshow (HL) χ^2^ statistics and calibration plots.

**Results:**

Multivariate logistic regression analyses revealed that female age, female body mass index (BMI), tubal factor infertility, male infertility, uterine factor infertility, unexplained infertility, antral follicle count (AFC) and basal serum follicle stimulating hormone (FSH) were significant factors for CLB in women undergoing the first IVF cycle. An area under the receiver operating characteristic curve (AUC) in the prediction model was 0.676 (95% CI 0.668 to 0.684) in the training group. The validation set showed possibly helpful discrimination with an AUC of 0.672 (95% CI 0.660 to 0.684). Additionally, the prediction model had a good calibration (HL χ^2^ = 8.240, P=0.410).

**Conclusions:**

We developed and validated a nomogram to predict CLB in women undergoing the first IVF cycle using a single center database in China. The validated nomogram to predict CLB could be a potential tool for IVF counselling.

## Introduction

Infertility is a high incidence in today’s society, accounting for about 10% to 15% of couples of reproductive age (1, 2). *In vitro* fertilization (IVF) has become a widespread option for treating fertility problems around the world (3). From the birth of the world’s first IVF baby in 1978 to the application of ovulation-stimulating drugs in IVF, IVF has moved from single follicular development to multiple follicles in each menstrual cycle, significantly increasing the pregnancy rate and live birth rate (4–6).

The success of IVF is conventionally defined as the live birth rate from a single fresh cycle. However, the widespread use of embryo cryopreservation over the past two decades, the cumulative live birth (CLB) rate, which includes frozen embryo replacements and subsequent treatment episodes, is more informative. Considering that many other factors can influence the success of IVF, it can be challenging for clinicians to counsel couples about their individualized chances of success. It can be difficult for clinicians to evaluate individualized chances of success before a complete IVF treatment. A clinical prediction model that could estimate the cumulative chances of a live birth before IVF begins would be essential for patient counseling and to help with IVF decision-making (7). There have been many attempts to build predictive models to predict IVF success, but most of them are about clinical pregnancy rate, ongoing pregnancy, or live birth rate (8–16). Predictive models of the CLB in women undergoing the first IVF cycle are limited. This study aims to develop and validate a nomogram for the CLB in women undergoing their first IVF cycle using a single-center database in China.

## Materials and methods

### Study design and participants

A cross-sectional study was conducted using 5–year clinical data (January 2014 to December 2018) from at Assisted Reproduction Center of Northwest Women’s and Children’s Hospital, Shaanxi province, Northwest China. A total 29,104 patients conceived with IVF/intracytoplasmic sperm injection (ICSI) treatment. Among these patients, we excluded 1,584 patients with missing pregnancy outcomes, and 831 patients with missing covariates (110 gravidity missing, 201 missing BMI, 15 antral follicles missing, 217 infertility duration missing, 288 basal follicle stimulating hormone (FSH) missing). Lastly, 26,689 patients were included in this study.

### Data collection and main variables

ART pregnancy outcomes (including clinical pregnancy and live birth) are required to be reported in the Assisted Reproduction Database in the Shaanxi province of China. In this study, CLB were collected from the Assisted Reproduction Database. Additionally, demographic data and ART treatment data were collected and assessed by each patient’s clinician.

Based on the literature (17), we collected all potential correlated factors of CLB from the records. These include female age, female BMI, gravidity, infertility duration, infertility type, tubal factor infertility (yes or no), ovarian factor infertility (yes or no), endometriosis infertility (yes or no), uterine factor infertility (yes or no), male factor infertility (yes or no), unexplained infertility (yes or no), antral follicle count (AFC) and basal FSH. Based on the previous study (18), participants were divided into five age groups: <25, 25–29, 30–34, 35–39 and ≥40 years. We selected 25–29 years as the reference age-group because of the greatest proportion of participants in the dataset was among the group of women with 25–29 years. Based on the Chinese criteria (19), participants were divided into four BMI groups: underweight (BMI < 18.5 kg/m^2^), normal weight (18.5 ≤ BMI < 24 kg/m^2^), overweight (24 ≤ BMI < 28 kg/m^2^) and obese (BMI ≥ 28 kg/m^2^). We selected normal weight group as the reference BMI-group. For gravidity, participants were divided into four gravidity groups: 0 (reference), 1, 2, and ≥ 3. For infertility duration, participants were divided into three groups: <2 (reference), 2–4, and ≥ 5 years. For infertility type, participants were divided into two groups: primary infertility (reference) and secondary infertility. AFC were classified by quartiles: ≤7 (reference), 8–11, 12–16, ≥17. And basal FSH were classified by quartiles: ≤5.70 (reference), 5.71–6.78, 6.79–8.20, ≥8.21 U/L.

The primary outcome was CLB, which was defined as at least one live birth resulting from one aspirated ART cycle in the fresh embryo transfer or subsequent frozen embryo transfer in relation to the number of oocytes retrieved. Only the first delivery was considered in the analysis. One treatment cycle is defined as an oocyte retrieval and all transfers, fresh and frozen/thawed, derived from that ovarian stimulation.

### Ethical approval

The Human Research Ethics Committee of the Northwest Women’s and Children’s Hospital approved this study in December 2019 (No. 2019013), and the committee waived the need to obtain informed consent in this study. All of the research was performed in accordance with the relevant guidelines and regulations.

### Statistical analysis

To generate nomograms and perform external verification, 70% subjects were randomly assigned to the training set, while the remaining 30% were assigned to the external validation set. Categorical variables were described as frequency (percentage) and the differences between groups were compared using the χ^2^ test.

In the training group, baseline variables that were considered clinically relevant or that showed a univariate relationship with CLB were entered into multivariate logistic regression model. All variables were tested for collinearity. Variables for inclusion were carefully chosen, given the outcome of forward stepwise method and the number of events available, to ensure parsimony of the final model. A nomogram was constructed based on the results of the multivariate logistic regression analysis and the selected variables were incorporated in the nomogram to predict the CLB. The nomogram is constructed by converting each regression coefficient in multivariate logistic regression to a scale of 0-100 points. 100 points are assigned to the variable with the highest β coefficient (absolute value). The points for each independent variable were added together to derive the total-point score for the predicted probability of the CLB. The model performance was evaluated using the C statistic, which is equivalent to the receiver operating characteristic curve (ROC) area under the receiver operating characteristic curve (AUC). Meanwhile, the calibration performance (agreement between observed and predicted frequencies of the CLB) was assessed by Hosmer-Lemeshow (HL) χ^2^ statistics and calibration plots. We also used decision curve analysis (DCA) to assess the net benefit of nomogram-assisted decisions.

Statistical analysis was performed using SPSS software (ver 23.0, USA), and R software (ver 3.4.1, USA). Two-tailed analysis with P<0.05 indicated that the difference was statistically significant.

## Results

### Participants’ characteristics

A total of 26,689 patients were enrolled, including 18,601 in the training group and 8,088 in the validation group. **Table 1** showed the baseline characteristics of the group. Comparison of the baseline data indicated that the training and validation groups showed no significant differences in the general situation of patients, years of infertility, type of infertility, cause of infertility, and the CLB rate.

**Table 1:** Basis characteristics of training group and validation group.

Variables	Training group (n=18601)	Validation group (n=8088)	χ^2^ value	*P* value
Female age (year), n (%)			8.351	0.074
<25	1123 (6.04)	471 (5.82)		
25–29	7332 (39.42)	3193 (39.48)		
30–34	6450 (34.68)	2704 (33.43)		
35–39	2631 (14.14)	1216 (15.03)		
≥40	1065 (5.73)	504 (6.23)		
Female BMI, n (%)			0.525	0.913
underweight	1619 (8.70)	712 (8.80)		
normal weight	11808 (63.48)	5105 (63.12)		
overweight	4093 (22.00)	1786 (22.08)		
obese	1081 (5.81)	485 (6.00)		
Gravidity, n (%)			0.885	0.829
0	10008 (53.80)	4323 (53.45)		
1	4224 (22.7)	1837 (22.71)		
2	2311 (12.42)	1002 (12.39)		
≥3	2058 (11.06)	926 (11.45)		
Infertility duration (year), n (%)			2.364	0.307
<2	3579 (19.24)	1590 (19.66)		
2–4	9900 (53.22)	4222 (52.20)		
≥5	5122 (27.54)	2276 (28.14)		
Infertility type, n (%)			0.570	0.450
primary infertility	10086 (54.22)	4345 (53.72)		
secondary infertility	8515 (45.78)	3743 (46.28)		
Tubal factor infertility, n (%)			0.127	0.721
yes	11627 (62.51)	5037 (62.28)		
no	6974 (37.49)	3051 (37.72)		
Ovarian factor, n (%)			0.104	0.748
yes	1197 (6.44)	529 (6.54)		
no	17404 (93.56)	7559 (93.46)		
Endometriosis infertility, n (%)			0.004	0.951
yes	891 (4.79)	386 (4.77)		
no	17710 (95.21)	7702 (95.23)		
Uterine factor infertility, n (%)			0.027	0.870
yes	1532 (8.24)	671 (8.30)		
no	17069 (91.76)	7417 (91.70)		
Male factor infertility, n (%)			0.001	0.973
yes	3285 (17.66)	1427 (17.64)		
no	15316 (82.34)	6661 (82.36)		
Unexplained infertility, n (%)			0.954	0.329
yes	3320 (17.85)	1484 (18.35)		
no	15281 (82.15)	6604 (81.65)		
AFC, n (%)			1.557	0.669
≤7	4708 (25.31)	2062 (25.49)		
8–11	4791 (25.76)	2026 (25.05)		
12–16	4864 (26.15)	2128 (26.31)		
≥17	4238 (22.78)	1872 (23.15)		
Basal FSH (U/L), n (%)			1.897	0.594
≤5.70	4630 (24.89)	2045 (25.28)		
5.71–6.78	4720 (25.37)	1989 (24.59)		
6.79–8.20	4634 (24.91)	2033 (25.14)		
≥8.21	4617 (24.82)	2021 (24.99)		
CLB, n (%)			1.516	0.218
yes	9453 (50.82)	4044 (50.00)		
no	9148 (49.18)	4044 (50.00)		

AFC and basal FSH were classified by quartiles.

### Nomogram development

The univariate associations of the potential predictors for CLB following IVF were shown in **Table 2**. By setting significance level to be 0.05, there are totally 13 statistically significant predictors: female age, female BMI, gravidity, infertility duration, infertility type, tubal factor infertility, ovarian factor infertility, endometriosis infertility, uterine factor infertility, male infertility, and unexplained infertility, AFC, Basal FSH.

**Table 2:** Univariate and multivariate logistic analysis of factors predicting CLB in the training group.

Variables	Live birth	Non-live birth	Univariate logistic analysis		Multivariate logistic analysis	
	(n=5453)	(n=5148)	OR (95%CI)	*P* value	OR (95%CI)	*P* value
Female age (year), n (%)						
<25	676 (60.20)	447 (39.80)	1.02 (0.90, 1.16)	0.731	0.91 (0.80, 1.04)	0.147
25–29	4374 (59.66)	2958 (40.34)	1.00		1.00	
30–34	3332 (51.66)	3118 (48.34)	0.72 (0.68, 0.77)	<0.001	0.83 (0.77, 0.89)	<0.001
35–39	935 (35.54)	1696 (64.46)	0.37 (0.34, 0.41)	<0.001	0.54 (0.49, 0.60)	<0.001
≥40	136 (12.77)	929 (87.23)	0.10 (0.08, 0.12)	<0.001	0.19 (0.16, 0.23)	<0.001
Female BMI, n (%)						
underweight	852 (52.63)	767 (47.37)	1.03 (0.93, 1.14)	0.591	0.98 (0.88, 1.09)	0.658
normal weight	6130 (51.91)	5678 (48.09)	1.00		1.00	
overweight	1978 (48.33)	2115 (51.67)	0.87 (0.81, 0.93)	<0.001	0.86 (0.80, 0.93)	<0.001
obese	493 (45.61)	588 (54.39)	0.78 (0.69, 0.88)	<0.001	0.69 (0.61, 0.79)	<0.001
Gravidity, n (%)						
0	5499 (54.95)	4509 (45.05)	1.00			
1	2127 (50.36)	2097 (49.64)	0.83 (0.77, 0.89)	<0.001		
2	1043 (45.13)	1268 (54.87)	0.67 (0.62, 0.74)	<0.001		
≥3	784 (38.10)	1274 (61.90)	0.51 (0.46, 0.56)	<0.001		
Infertility duration (year), n (%)						
<2	1792 (50.07)	1787 (49.93)	1.00			
2–4	5284 (53.37)	4616 (46.63)	1.14 (1.06, 1.23)	0.001		
≥5	2377 (46.40)	2745 (53.59)	0.86 (0.79, 0.94)	0.001		
Infertility type, n (%)						
primary infertility	5535 (54.88)	4551 (45.12)	1.00			
secondary infertility	3918 (46.01)	4597 (53.99)	0.70 (0.66, 0.74)	<0.001		
Tubal factor infertility, n (%)						
yes	5986 (51.48)	5641 (48.52)	1.07 (1.01, 1.14)	0.019	1.30 (1.20, 1.41)	<0.001
no	3467 (49.71)	3507 (50.29)	1.00		1.00	
Ovarian factor infertility, n (%)						
yes	482 (40.27)	715 (59.73)	0.63 (0.56, 0.71)	<0.001		
no	8971 (51.55)	8433 (48.45)	1.00			
Endometriosis infertility, n (%)						
yes	416 (46.69)	475 (53.31)	0.84 (0.73, 0.96)	0.012		
no	9037 (51.03)	8673 (48.97)	1.00			
Uterine factor infertility, n (%)						
yes	629 (41.06)	903 (58.94)	0.65 (0.59, 0.72)	<0.001	0.88 (0.78, 0.99)	0.029
no	8824 (51.70)	8245 (48.30)	1.00		1.00	
Male factor infertility, n (%)						
yes	1991 (60.61)	1294 (39.39)	1.62 (1.50, 1.75)	<0.001	1.40 (1.29, 1.53)	<0.001
no	7462 (48.72)	7854 (51.28)	1.00		1.00	
Unexplained infertility, n (%)						
yes	1857 (55.93)	1463 (44.07)	1.28 (1.19, 1.39)	<0.001	1.20 (1.08, 1.33)	0.001
no	7596 (49.71)	7685 (50.29)	1.00		1.00	
AFC, n (%)						
≤7	1400 (29.74)	3308 (70.26)	1.00		1.00	
8–11	2483 (51.83)	2308 (48.17)	2.54 (2.34, 2.77)	<0.001	1.86 (1.70, 2.03)	<0.001
12–16	2954 (60.73)	1910 (39.27)	3.65 (3.36, 3.98)	<0.001	2.34 (2.13, 2.57)	<0.001
≥17	2616 (61.73)	1622 (38.27)	3.81 (3.49, 4.16)	<0.001	2.41 (2.18, 2.66)	<0.001
Basal FSH (U/L), n (%)						
≤5.70	2649 (57.21)	1981 (42.79)	1.00		1.00	
5.71–6.78	2669 (56.55)	2051 (43.45)	0.97 (0.90, 1.06)	0.515	0.99 (0.91, 1.08)	0.884
6.79–8.20	2415 (52.11)	2219 (47.89)	0.81 (0.75, 0.88)	<0.001	0.89 (0.82, 0.97)	0.010
≥8.21	1720 (37.25)	2897 (62.75)	0.44 (0.41,0.48)	<0.001	0.64 (0.59, 0.70)	<0.001

The multivariate logistic regression model predicting CLB was also displayed in **Table 2**. The model showed that the odds of a successful CLB decrease with older age (30–34 vs 25–29 years: OR=0.83, 95%CI=0.77, 0.89; 35–39 vs 25-29 years: OR=0.54, 95%CI=0.49, 0.60; ≥40 vs 25–29 years: OR=0.19, 95%CI=0.16, 0.23), overweight or obese (overweight vs normal weight: OR=0.86, 95%CI=0.80, 0.93; obese vs normal weight: OR=0.69, 95%CI=0.61, 0.79), uterine factor infertility (OR=0.88, 95%CI=0.78, 0.99) and higher basal FSH (6.79–8.20 vs ≤5.70: OR=0.89, 95%CI=0.82, 0.97; ≥8.21 vs ≤5.70: OR=0.64, 95%CI=0.59, 0.70). Other variables which showed a statistically significant increase in odds of CLB in the final model were: tubal factor infertility (OR=1.30, 95%CI=1.20, 1.41), male infertility (OR=1.40, 95%CI=1.29, 1.53), unexplained infertility (OR=1.20, 95%CI=1.08, 1.33) and higher AFC (8–11 vs ≤7: OR=1.86, 95%CI=1.70, 2.03; 12–16 vs ≤7: OR=2.34, 95%CI=2.13, 2.57; ≥17 vs ≤7: OR=2.41, 95%CI=2.18, 2.66).

**Figure 1:**
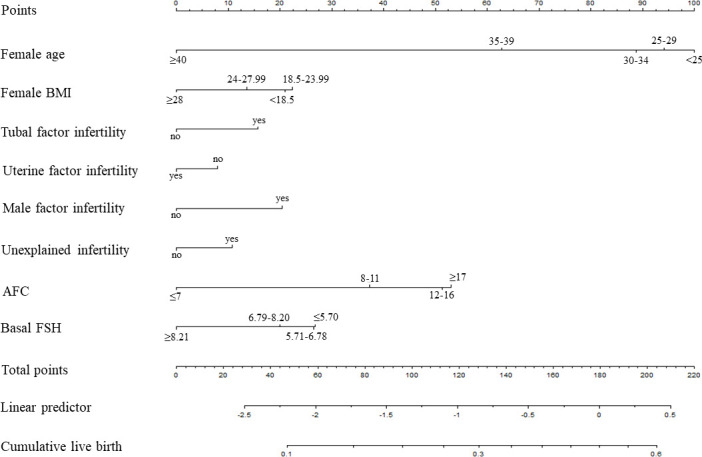
Nomogram for predicting CLB.

**Figure 2:**
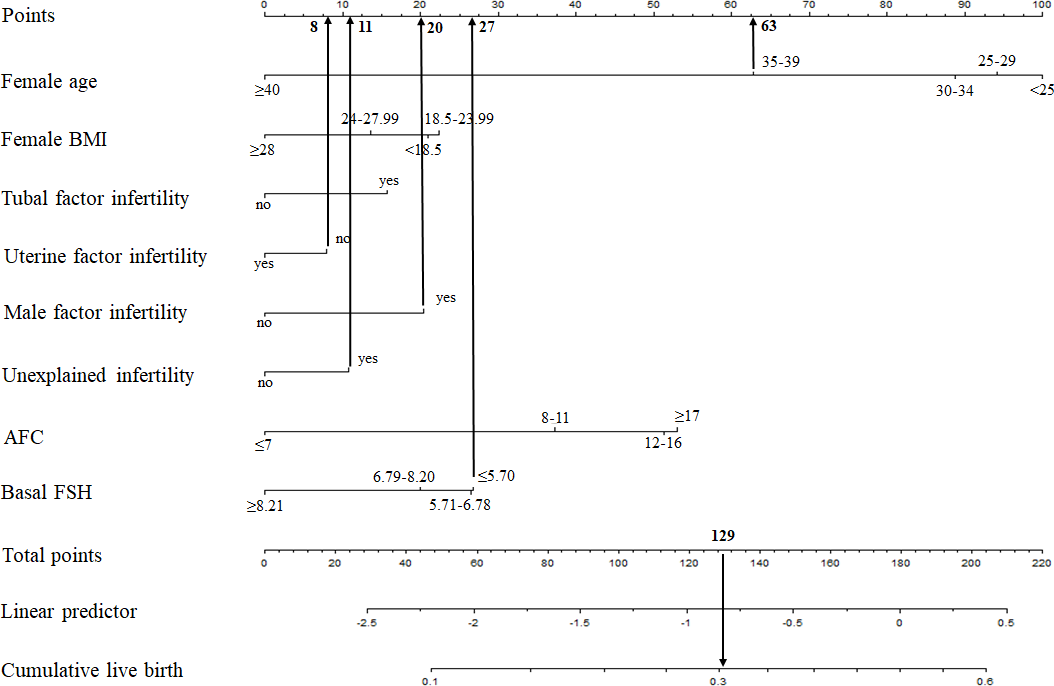
Example prediction nomogram for risk of CLB. A couple with ART treatment: female age=36 years (63 points), female BMI= 30 kg/m^2^ (0 points), no tubal factor infertility (0 points), no uterine factor infertility (8 points), male factor infertility (20 points), unexplained infertility (11 points), AFC=5 (0 points), basal FSH=3.67 U/L (27 points). The cumulative score of the various prediction indicators was 63 + 8 + 20 + 11 + 27 = 129, and the corresponding predicted risk of CLB was 0.30 (30%).

Based on the multivariate logistic regression analysis, the eight independent predictors were included in the prediction model. We then establish an individualized nomogram prediction model of CLB following IVF (**Figure 1**). The application of the nomogram is as follows: based on the nomogram, we can obtain the points corresponding to each prediction indicator, the sum of the points is recorded as the total score, and the predicted risk corresponding to the total score is the probability of CLB following IVF (**Figure 2**).

### Nomogram validation

**Figure 3:**
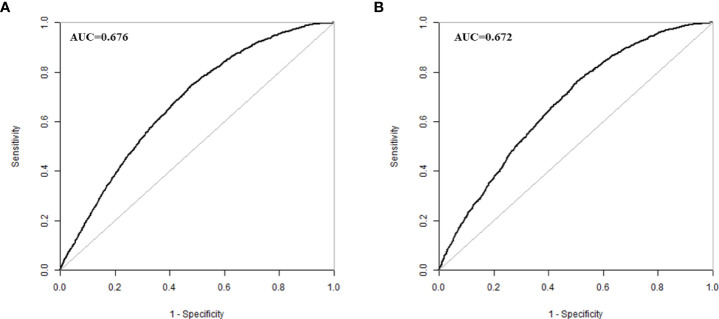
ROC curves in training group **(A)** and validation group **(B)**.

**Figure 4:**
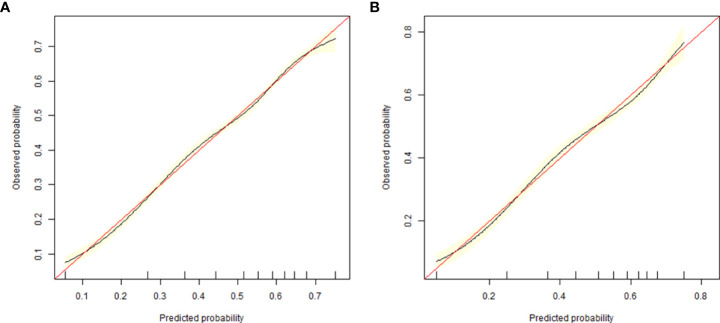
Calibration curves of the nomogram in training group **(A)** and validation group **(B)**.

**Figure 5:**
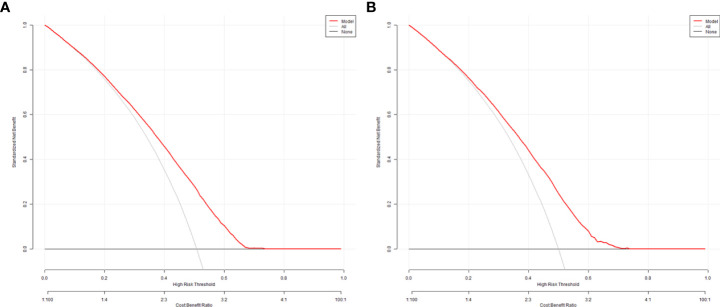
DCA curves of the nomogram in training group **(A)** and validation group **(B)**.

The validation of the model was based on discrimination and calibration. We drew the ROC curves of predicted probability and calculated the AUC values in the training group and validation group (20). The ROC curve was used to compute the AUC values from models with the eight independent predictors in the nomogram. The AUC values of the training group and validation group were 0.676 (95%CI=0.668, 0.684) and 0.672 (95%CI=0.660, 0.684) (**Table 3**, **Figure 3**) respectively, suggesting that the nomogram prediction model had a possibly helpful discrimination. The HL χ^2^ statistics was 8.240 (P=0.410) and the calibration plots, which revealed the prediction model had a good calibration. In the training group and validation group, calibration curves swung around the 45-degree oblique line, indicating a high degree of calibration (**Figure 4**). The decision curves for CLB probability showed that the net income of the training and validation groups was higher when the probability was between 50% and 70% (**Figure 5**).

**Table 3:** The AUCs of the ROC curves for the nomogram and variables from the logistic regression model in the training group and validation group.

Variables	Development group			Validation group		
	AUC	95%CI	P value	AUC	95%CI	P value
Nomogram variable	0.676	0.668, 0.684	<0.001	0.672	0.660, 0.684	<0.001
Female age	0.616	0.608, 0.624	<0.001	0.620	0.608, 0.624	<0.001
Female BMI	0.519	0.510, 0.527	<0.001	0.514	0.502, 0.527	0.026
Tubal factor infertility	0.508	0.500, 0.517	0.049	0.511	0.499, 0.524	0.080
Uterine factor infertility	0.516	0.508, 0.524	<0.001	0.515	0.503, 0.528	0.016
Male factor infertility	0.535	0.526, 0.543	<0.001	0.531	0.518, 0.543	<0.001
Unexplained infertility	0.518	0.510, 0.527	<0.001	0.519	0.506, 0.532	0.003
AFC	0.632	0.624, 0.640	<0.001	0.634	0.622, 0.646	<0.001
Basal FSH	0.580	0.572, 0.588	<0.001	0.572	0.559, 0.584	<0.001

## Discussion

Successful prediction of CLB after IVF has been limited. This study of 26,689 IVF patients is, to our knowledge, the larger sample of studies to evaluate the CLB of IVF patients, leading to the development of a prediction model to calculate cumulative lives’ chances of birth after IVF. The critical predictors in our model that were shown to affect CLB rate are female age, female BMI, tubal factor infertility, uterine factor, male infertility, unexplained infertility, AFC and basal FSH.

A model has been developed that clinician may use before IVF treatment to estimate a couple’s chances of having a live birth over one complete cycle of IVF. Before IVF treatment, only information about the couple at that time can be used to predict the outcome. Therefore, when clinicians will use the pre-IVF model to counsel couples as to their future chances of success, the female age and BMI, cause of infertility, AFC and basal FSH are known. We acknowledge that information from future embryo transfer cycles would provide more precise predictions, but our model reflects a more real life setting where such information is unavailable at the time of counselling. This model will provide an indication of the couple’s future chances of achieving a live birth over a complete cycle of IVF before the beginning of treatment.

There have been some predictive models about the CLB rate after IVF (21, 22). One is a retrospective cohort study reported by Balachandren et al. in 2020 (21). Though this prediction model estimated a couple’s individualized probability of achieving a live birth after their first complete IVF cycle using all known pre-treatment predictors, it only included 516 complete IVF cycles. A systematic review and meta-analysis on predictive factors in IVF evaluated nine predictive factors: female age, length of infertility, type of infertility, indication for IVF, basal FSH, fertilization method, number of oocytes, number of embryos transferred, and embryo quality (17). This model is for pre-treatment model only, we did not include any oocyte or embryo factors. We believe this prediction model holds an important role in the counselling process for women before they embark on the first IVF cycle (23). The calculated probabilities are expressed per couple and not per cycle.

Many factors affect the CLB of IVF, among which female age accounts for a large proportion. During IVF treatment, the effects of female age on CLB are mainly manifested as decreased ovarian responsiveness to ovulation-stimulating drugs, decreased oocyte quality, low embryo implantation rate, increased abortion rate, and decreased delivery rate (24). Data from the US Centers for Disease Control and Prevention (CDC) in 2010 showed that: in the 147,260 non-donor egg fresh IVF cycles, the pregnancy rate of women younger than 34 years old was relatively stable and showed a linear downward trend after 35 years old, and it dropped to zero at 44 years old (25). A meta-analysis in 2013 showed that among women who received ART treatment, for every one year of age increase, the pregnancy rate decreased by 6% (26). Malizia et al. used the 14,248 cycles of 6,164 patients to accumulate the live birth rate of 6 oocyte retrieval cycles and found that: IVF can enable most young women to obtain live births, but it cannot reverse the decline in pregnancy rate brought about by age (27).

Ovarian reserve markers such as AFC, anti-Mullerian hormone (AMH), and basal FSH are also key predictors of CLB (28). Systematic reviews have suggested that AFC and AMH were the best predictors of excessive and suboptimal ovarian response (29, 30). Li, HW et al. found that women who attained a CLB had significantly higher serum AMH and AFC at baseline before ovarian stimulation (31). In consideration of saving costs for patients, Assisted Reproduction Center of Northwest Women’s and Children’s Hospital would not test AMH unless it is necessary. Due to incomplete AMH data, we only included AFC and basal FSH in this study. There are cyclical fluctuations in basal FSH, and different centers have different reference ranges. Most of them use basal FSH level>12 U/L as an indicator of decreased ovarian reserve (32). At the same age, the ovarian reserve and responsiveness are also different, so it is necessary to combine age, AFC, basal FSH to predict CLB rate.

Although we have produced good results, the current study still had several limitations. Firstly, because this study is a cross-sectional study, 2,416 (8.30%) of the 29,104 patients were excluded, which can lead to selection bias. Secondly, although we investigated as many CLB-related risk factors as possible, some unmeasured predictors may have been ignored because data in the hospital information system were limited. For example, AMH is missing in this prediction model, which is an important indicator in assessing ovarian function. Therefore, the lack of some important predictors may affect the prediction model’s performance. Thus, further improvement of the prediction model could be completed by adding more prognostic factors in future studies. Lastly, the data for the prediction model was derived from a single center in Shaanxi province, Northwest China, and the assisted reproduction center of Northwest Women’s and Children’s Hospital mainly covers the Shaanxi province and surrounding areas. Given that absolute risks are highly sensitive to the distribution of each predictor in the study population. Therefore, our prediction model may apply to women contemplating ART in Shaanxi province and surrounding areas. For the generalizability of our prediction model to other populations, we still need evidence from other centers for validation. Therefore, in the follow-up research work, we will persuade other medical centers to join this research project and provide the appropriate clinical data to conduct a more in-depth assessment and validation of the prediction model.

## Conclusion

In summary, we established an individualized nomogram for the CLB in women undergoing their first IVF cycle. Through this prediction model, we can accurately predict the CLB rate based on each patient’s characteristics, which could be a potential tool for IVF counselling, and further treatment can also be decided based on the result of this nomogram. Further prospective validation studies with multicenter should be undertaken to confirm the efficacy of the application of the current nomogram to IVF patients.

## Data availability statement

The raw data supporting the conclusions of this article will be made available by the authors, without undue reservation.

## Ethics statement

The studies involving human participants were reviewed and approved by The Human Research Ethics Committee of the Northwest Women’s and Children’s Hospital. Written informed consent for participation was not required for this study in accordance with the national legislation and the institutional requirements.

## Author contributions

PQ, WS, and JS conceived and designed the study. PQ, LC, WS and JS drafted and revised the manuscript. PQ and DZ analyzed and interpreted the data. PQ, LC and WS collected and cleared the data. All authors have read and approved the final version of the manuscript.

## Funding

This work was supported by the National Natural Science Foundation of China (No. 82103924) and the Key Research and Development Program of Shaanxi Province (No. 2021ZDLSF02-14; 2022ZDLSF02-11).

## Acknowledgments

We thank the staff from Northwest Women’s and Children’s Hospital for their assistance with the data collection. We thank all participants in this study.

## Conflict of interest

The authors declare that the research was conducted in the absence of any commercial or financial relationships that could be construed as a potential conflict of interest.

## Publisher’s note

All claims expressed in this article are solely those of the authors and do not necessarily represent those of their affiliated organizations, or those of the publisher, the editors and the reviewers. Any product that may be evaluated in this article, or claim that may be made by its manufacturer, is not guaranteed or endorsed by the publisher.

## References

[1] Thoma ME Mclain AC Louis JF King RB Trumble AC Sundaram R. Prevalence of infertility in the united states as estimated by the current duration approach and a traditional constructed approach. Fertil Steril (2013) 99(5):1324–31. doi: 10.1016/j.fertnstert.2012.11.037 PMC3615032 23290741

[2] Mascarenhas MN Flaxman SR Boerma T Vanderpoel S Stevens GA. National, regional, and global trends in infertility prevalence since 1990: A systematic analysis of 277 health surveys. PLoS Med (2012) 9(12):e1001356. doi: 10.1371/journal.pmed.1001356 23271957 PMC3525527

[3] de Mouzon J Chambers GM Zegers-Hochschild F Mansour R Ishihara O Banker M. International committee for monitoring assisted reproductive technologies world report: Assisted reproductive technology 2012 †. Hum Reprod (2020) 35(8):1900–13. doi: 10.1093/humrep/deaa090 32699900

[4] Steptoe PC Edwards RG. Birth after the reimplantation of a human embryo. Lancet (1978) 2(8085):366. doi: 10.1016/s0140-6736(78)92957-4 79723

[5] Trounson AO Leeton JF Wood C Webb J Wood J. Pregnancies in humans by fertilization *in vitro* and embryo transfer in the controlled ovulatory cycle. Science (1981) 212(4495):681–2. doi: 10.1126/science.7221557 7221556

[6] Briggs R Kovacs G MacLachlan V Motteram C Baker HW. Can you ever collect too many oocytes? Hum Reprod (2015) 30(1):81–7. doi: 10.1093/humrep/deu272 25362088

[7] van der Steeg JW Steures P Eijkemans MJ Habbema JD Bossuyt PM Hompes PG. Do clinical prediction models improve concordance of treatment decisions in reproductive medicine? BJOG (2006) 113(7):825–31. doi: 10.1111/j.1471-0528.2006.00992.x 16827767

[8] Hunault CC Eijkemans MJ Pieters MH te Velde ER Habbema JD Fauser BC. A prediction model for selecting patients undergoing *in vitro* fertilization for elective single embry o transfer. Fertil Steril (2002) 77(4):725–32. doi: 10.1016/s0015-0282(01)03243-5 11937124

[9] van Weert JM Repping S van der Steeg JW Steures P van der Veen F Mol BW. A prediction model for ongoing pregnancy after *in vitro* fertilization in couples with male subfertility. J Reprod Med (2008) 53(4):250–6. 18472647

[10] Lintsen AM Eijkemans MJ Hunault CC Bouwmans CA Hakkaart L Habbema JD. Predicting ongoing pregnancy chances after IVF and ICSI: A national prospective study. Hum Reprod (2007) 22(9):2455–62. doi: 10.1093/humrep/dem183 17636281

[11] La Marca A Nelson SM Sighinolfi G Manno M Baraldi E Roli L. Anti-mülleria hormone-based prediction model for a live birth in assisted reproduction. Reprod BioMed Online (2011) 22(4):341–9. doi: 10.1016/j.rbmo.2010.11.005 21317041

[12] Porcu G Lehert P Colella C Giorgetti C. Predicting live birth chances for women with multiple consecutive failing IVF cycles: A simple and ac curate prediction for routine medical practice. Reprod Biol Endocrinol (2013) 11:1. doi: 10.1186/1477-7827-11-1 23302328 PMC3551786

[13] Veltman-Verhulst SM Fauser BC Eijkemans MJ. High singleton live birth rate confirmed after ovulation induction in women with anovulatory polycyst ic ovary syndrome: Validation of a prediction model for clinical practice. Fertil Steril (2012) 98(3):761–768.e1. doi: 10.1016/j.fertnstert.2012.04.027 22633255

[14] Luke B Brown MB Wantman E Stern JE Baker VL Widra E. A prediction model for live birth and multiple births within the first three cycles of assisted repro ductive technology. Fertil Steril (2014) 102(3):744–52. doi: 10.1016/j.fertnstert.2014.05.020 PMC4188433 24934487

[15] Nelson SM Lawlor DA. Predicting live birth, preterm delivery, and low birth weight in infants born from *in vitro* fertilisa tion: A prospective study of 144,018 treatment cycles. PLoS Med (2011) 8(1):e1000386. doi: 10.1371/journal.pmed.1000386 21245905 PMC3014925

[16] te Velde ER Nieboer D Lintsen AM Braat DD Eijkemans MJ Habbema JD. Comparison of two models predicting IVF success; the effect of time trends on model performance. Hum Reprod (2014) 29(1):57–64. doi: 10.1093/humrep/det393 24242632

[17] Van, Loendersloot L,L Van, Wely,M Limpens J Bossuyt PMM. Predictive factors in *in vitro* fertilization (IVF): A systematic review and meta-analysis. Hum Reprod Update (2010) 16(6):577–89. doi: 10.1093/humupd/dmq015 20581128

[18] Cavazos-Rehg PA Krauss MJ Spitznagel EL Bommarito K Madden T Olsen MA. Maternal age and risk of labor and delivery complications. Matern Child Health J (2015) 19(6):1202–11. doi: 10.1007/s10995-014-1624-7 PMC4418963 25366100

[19] National Health and Family Planning Commission of the People's Republic of China. Criteria of weight for adults. Health industry standard of the People's Republic of China. (2013) WS/T 428–2013.

[20] Harrell FE Califf RM Pryor DB Lee KL Rosati RA. Evaluating the yield of medical tests. JAMA (1982) 247(18):2543–6 doi: 10.1001/jama.1982.03320430047030. 7069920

[21] Balachandren N Salman M Diu NL Schwab S Rajah K Mavrelos D. Ovarian reserve as a predictor of cumulative live birth. Eur J Obstet Gynecol Reprod Biol (2020) 252:273–7. doi: 10.1016/j.ejogrb.2020.06.063 32645642

[22] Leijdekkers JA Eijkemans MJC van Tilborg TC Oudshoorn SC McLernon DJ Bhattacharya S. Predicting the cumulative chance of live birth over multiple complete cycles of *in vitro* fertilization: An external validation study. Hum Reprod (2018) 33(9):1684–95. doi: 10.1093/humrep/dey263. O. group. 30085143

[23] Dhillon RK McLernon DJ Smith PP Fishel S Dowell K Deeks JJ. Predicting the chance of live birth for women undergoing IVF: A novel pretreatment counselling tool. Hum Reprod (2016) 31(1):84–92. doi: 10.1093/humrep/dev268 26498177

[24] M. Practice Committee of the American Society for Reproductive. Aging and infertility in women. Fertil Steril (2006) 86(5 Suppl 1):S248–52. doi: 10.1016/j.fertnstert.2006.08.024 17055834

[25] Sunderam S Kissin DM Crawford S Anderson JE Folger SG Jamieson DJ. Division of reproductive health, national center for chronic disease prevention and health promotion, CDC: Assisted reproductive technology surveillance – united states, 2010. MMWR Surveill Summ (2013) 62(9):1–24. 24304902

[26] Broer SL van Disseldorp J Broeze KA Dolleman M Opmeer BC Bossuyt P. Added value of ovarian reserve testing on patient characteristics in the prediction of ovarian respon se and ongoing pregnancy: An individual patient data approach. Hum Reprod Update (2013) 19(1):26–36. doi: 10.1093/humupd/dms041 23188168

[27] Malizia BA Hacker MR Penzias AS. Cumulative live-birth rates after *in vitro* fertilization. N Engl J Med (2009) 360(3):236–43. doi: 10.1056/NEJMoa0803072 19144939

[28] Drakopoulos P Blockeel C Stoop D Camus M de Vos M Tournaye H. Conventional ovarian stimulation and single embryo transfer for IVF/ICSI. How many oocytes do we need to maximize cumulative live birth rates after utilization of all fresh and frozen embryos? Hum Reprod (2016) 31(2):370–6. doi: 10.1093/humrep/dev316 26724797

[29] Broer SL Mol BW Hendriks D Broekmans FJ. The role of antimullerian hormone in prediction of outcome after IVF: Comparison with the antral foll icle count. Fertil Steril (2009) 91(3):705–14. doi: 10.1016/j.fertnstert.2007.12.013 18321493

[30] Broer SL D¨®lleman M Opmeer BC Fauser BC Mol BW Broekmans FJ. AMH and AFC as predictors of excessive response in controlled ovarian hyperstimulation: a meta-analys is. Hum Reprod Update (2011) 17(1):46–54. doi: 10.1093/humupd/dmq034 20667894

[31] Li HWR Lee VCY Lau EYL Yeung WSB Ho P-C Ng EHY. : Role of baseline antral follicle count and anti-mullerian hormone in prediction of cumulative live birth in the first in vitro fertilisation cycle: A retrospective cohort analysis. PLoS One (2013) 8(4):e61095. doi: 10.1371/journal.pone.0061095.t001 23637787 PMC3634063

[32] Kofinas JD Elias RT. Follicle-stimulating hormone/luteinizing hormone ratio as an independent predictor of response to con trolled ovarian stimulation. Womens Health (Lond) (2014) 10(5):505–9. doi: 10.2217/whe.14.31 24807379

